# A Precautionary Approach to Guide the Use of Transition Metal-Based Nanotechnology to Prevent Orthopedic Infections

**DOI:** 10.3390/ma12020314

**Published:** 2019-01-20

**Authors:** Marta Bottagisio, Arianna B. Lovati, Fabio Galbusera, Lorenzo Drago, Giuseppe Banfi

**Affiliations:** 1 IRCCS Orthopedic Institute Galeazzi, Laboratory of Clinical Chemistry and Microbiology, Via R. Galeazzi 4, 20161 Milan, Italy; 2 IRCCS Orthopedic Institute Galeazzi, Cell and Tissue Engineering Laboratory, Via R. Galeazzi 4, 20161 Milan, Italy; arianna.lovati@grupposandonato.it; 3 IRCCS Orthopedic Institute Galeazzi, Laboratory of Biological Structures Mechanics, Via R. Galeazzi 4, 20161 Milan, Italy; fabio.galbusera@grupposandonato.it; 4 Laboratory of Clinical Microbiology, Department of Biomedical Sciences for Health, University of Milan, 20133 Milan, Italy; lorenzo.drago@unimi.it; 5 IRCCS Orthopedic Institute Galeazzi, Laboratory of Experimental Biochemistry & Molecular Biology, Via R. Galeazzi 4, 20161 Milan, Italy; banfi.giuseppe@fondazionesanraffaele.it; 6 Vita-Salute San Raffaele University, 20132 Milan, Italy

**Keywords:** nanoparticles, orthopedic infections, transition metals, antibacterial coatings, biofilm, antibiotic-resistant microorganisms

## Abstract

The increase of multidrug-resistant bacteria remains a global concern. Among the proposed strategies, the use of nanoparticles (NPs) alone or associated with orthopedic implants represents a promising solution. NPs are well-known for their antimicrobial effects, induced by their size, shape, charge, concentration and reactive oxygen species (ROS) generation. However, this non-specific cytotoxic potential is a powerful weapon effective against almost all microorganisms, but also against eukaryotic cells, raising concerns related to their safe use. Among the analyzed transition metals, silver is the most investigated element due to its antimicrobial properties *per se* or as NPs; however, its toxicity raises questions about its biosafety. Even though it has milder antimicrobial and cytotoxic activity, TiO_2_ needs to be exposed to UV light to be activated, thus limiting its use conjugated to orthopedic devices. By contrast, gold has a good balance between antimicrobial activity as an NP and cytocompatibility because of its inability to generate ROS. Nevertheless, although the toxicity and persistence of NPs within filter organs are not well verified, nowadays, several basic research on NP development and potential uses as antimicrobial weapons is reported, overemphasizing NPs potentialities, but without any existing potential of translation in clinics. This analysis cautions readers with respect to regulation in advancing the development and use of NPs. Hopefully, future works in vivo and clinical trials will support and regulate the use of nano-coatings to guarantee safer use of this promising approach against antibiotic-resistant microorganisms.

## 1. Introduction

Multidrug-resistant (MDR) bacteria remain a global concern, resulting in infectious diseases that are more and more difficult to treat [1]. The development of antibiotic resistance is related to several key factors correlated with the misuse of antibiotics: (1) the overuse of antibiotics; (2) the inappropriate or suboptimal prescription of these drugs reported in 30–50% of cases [1]; (3) the lack of information and education, which lead patients to prematurely interrupt the antibiotic course; the purchase of antibiotics without a medical prescription [2]; and (4) the decline of investments in new drug development in the pharmaceutical industry due to marketing concerns [3].

In orthopedics, the implantation of devices (e.g., prosthesis, plate, and screws, etc.) establishes a non-negligible incidence of infections, representing one of the major causes of morbidity and mortality in this medical field [4,5]. Despite prophylaxis, it has been estimated that 0.4–2% of patients develop this harmful complication following primary implantation and 5–15% after revision surgery [6,7,8]. The presence of a foreign body is the triggering event for implant-associated infections, because the surface and roughness of these biomaterials not only attract the host eukaryotic cells involved in the regeneration of tissues, but also free-floating bacteria. Indeed, as soon as a contamination occurs, the “race to the surface” begins, as first described by Gristina and colleagues, determining the fate of the development of the infection [9]. If the race to the surface is won by cells of the surrounding tissue, the implant surface will be occupied and, therefore, defended. Otherwise, bacteria rapidly adhere to the biomaterial and colonize the surface due to several physicochemical interactions (e.g., van der Waals and gravitational forces, electrostatic repulsion, and ionic and dipole interactions). Thereafter, bacteria start to proliferate and to aggregate in clusters through cell-to-cell adhesion. Guided by molecular signals, they secrete an extracellular polymeric matrix to form a multi-layered biofilm [10,11]. Biofilms enable bacteria to live in a protected environment with a renewable nutrient supply, without being affected by the physical forces associated with the fluid stream or by the host immune system [12]. Under such conditions, the minimum inhibitory concentration (MIC) of antimicrobial substances is 10–10^3^ times higher, thus enhancing the development of resistance in bacterial communities [13]. Hence, minimizing the risk of implant-related infections, emphasizing prophylaxis measures while discouraging the impairment of bone healing become of critical importance. 

To face the problem, different strategies have been proposed and pursued, such as the modification of the existing antimicrobial substances or the development of more effective ones to overcome bacterial resistance [14]. Among new perspectives proposed to combat and defeat both implant-associated infections and the rise of antimicrobial resistance, the use of nanoparticles (NPs) alone or associated with orthopedic implants as coatings represents a possible and promising solution. In particular, nano-sized transition metals (e.g., silver, gold, copper, zinc oxide, titanium dioxide, etc.,) demonstrate a versatile and controllable application in the fight against infections and antibiotic resistance development. Indeed, this strategy exploits the ability of NPs to cause bacterial damages at the molecular level, due to their ultra-small dimensions increasing the biophysical interaction with bacteria and the generation of free radicals [15]. Furthermore, metallic NPs play a crucial role in the prevention of biofilm formation, including Ag NPs, Au NPs, ZnO NPs, CuO NPs, Fe_3_O_4_ NPs [16,17,18]. As aforementioned, a smaller size and higher surface area-to-mass ratio are the performance-enhancing factors, but the shape of metal NPs also has a remarkable effect against biofilms [19].

Many concerns have been expressed by the scientific community whether the extremely active NPs might be a threat for bone cells surrounding the implanted devices and for tissues and organs. Indeed, a negative influence of NPs on eukaryotic cells like osteoblasts, osteoclasts, and bone marrow mesenchymal stem cells might result in an impairment in the implant integration in the absence of a bacterial infection. Fortunately, nowadays, nanotechnology offers novel materials able to support the host tissue function, favoring osteoblast attachment, proliferation, and synthesis of the extracellular matrix and enabling the osseointegration of the implant [20]. 

In this context, nanomaterials can be considered strong candidates in the control of resistant bacterial infections, limiting the consumption of antibiotics. Hence, the aim of this review is to describe different inorganic transition metal-based NPs and their antimicrobial and possible cytotoxic activity alone or conjugated with implantable materials to elucidate their promising role in orthopedics.

## 2. Antibacterial Properties of NPs: Mechanisms of Action

Metal and metal oxide NPs are well-known for their antimicrobial effects and, their extensive use in several clinical and industrial setting dates back to several decades ago [5]. 

**Figure 1:**
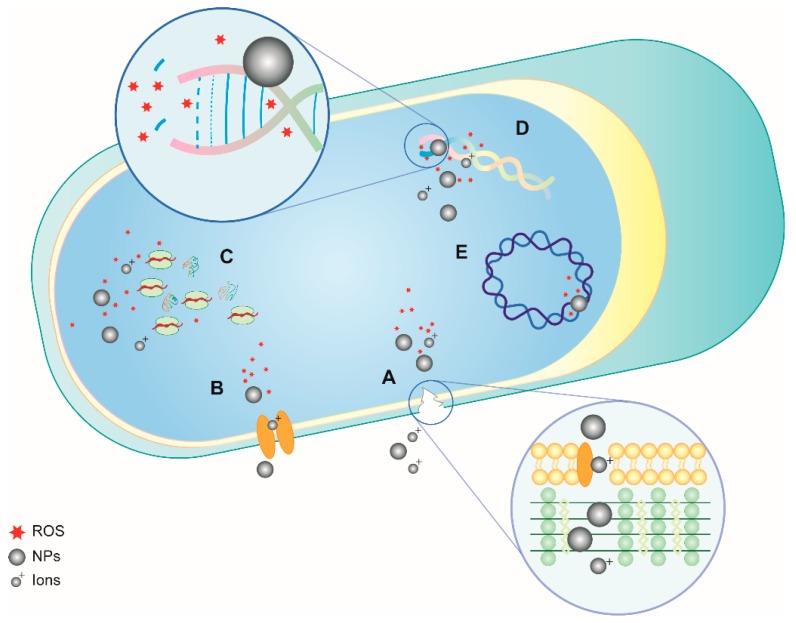
Schematic representation of the reaction of bacteria following the exposure to reactive oxygen species generated by NPs and ions. The illustration shows (**A**) cell wall disruption and NP and ion penetration, (**B**) the interruption of electron transport, (**C**) damages to the ribosome affecting the protein synthesis, (**D**) intercalation between DNA bases with consequent irreparable damages, and (**E**) the negative interaction with plasmid DNA.

Although not all the transition metal NPs share the same mechanisms of action, the properties of the majority of NPs are related to both their physical structure and to their specific interaction with biofilm producers. Several factors might induce and promote antimicrobial activity, like the (1) size and (2) shape of NPs. Indeed, as the NP size decreases, not only does their stability increase, but also the surface/volume ratio, conferring them a higher ability to interact with the cell membrane and consequently to have higher antimicrobial potential [21,22]. This interaction is also possible due to the key role played by the (3) electrostatic forces guiding bacterial and NP attraction. Indeed, most bacteria have a negatively charged cell wall that attracts positively charged molecules [23]. Positively charged ions or NPs can easily enter microorganisms, damaging their inner structures by binding negatively charged proteins and nucleic acids [24]. Furthermore, similar to any other antimicrobial agent, the bactericidal effect of NPs depends on the concentration (4), which can vary based on different bacterial susceptibility depending on the different microorganism classes. All the listed variables cooperate to confer NPs an antimicrobial effect amplified by the release of ions (5) [25]. Nevertheless, the (6) generation of reactive oxygen species (ROS) plays a crucial role in the bactericidal effect of NPs. Indeed, the local production of oxygen-free radicals leads to peroxidation of lipids, alteration at proteomic and enzymatic levels and also damages RNA and DNA (Figure 1) [5]. This cytotoxic and genotoxic potential is a powerful, non-specific weapon effective against almost all the type of microorganisms and also eukaryotic cells, raising many concerns related to the biocompatibility of NPs. Indeed, even if mammalian cells are able to limit the free-radical damage when ROS production exceeds this capability, it results in oxidative stress, inflammation, and irreparable damage to membranes, proteins, and DNA. Hence, to avoid any dangerous effects on eukaryotic cells, it is important to respect the concentration window that regulates the use of NPs in order to kill microorganisms without detrimental effects on osteoblasts and other host cells [26].

## 3. Transition Metal NPs with Antimicrobial Activity for Potential Use in Orthopedics

### 3.1. Silver

Silver (Ag) has long been recognized for its antibacterial properties. Ag antiseptic and antimicrobial features against Gram-positive, Gram-negative bacteria and fungi, indeed, date back to several decades ago [27,28]. Nowadays, Ag is frequently used in many different forms and is applied in several medical fields, among them in wound or burns as dressings, creams, or spray and as a coating on implantable devices. Due to the lack of mechanical strength, Ag is not currently employed as a bulk material for the production of orthopedic implants. However, Ag is often employed in the modification of the surface of some specific implants called “megaendoprosthesis”, used in the treatment of bone tumors or in the case of revision surgeries of septic devices [29]. Indeed, the ions released from the Ag-coated prosthesis demonstrated a high and broad-spectrum antibacterial effect, necessary for the prevention of infections associated with implantable devices in subjects with a higher risk factor, such as immunocompromised, oncologic or elderly patients. 

Silver nanoparticles (Ag NPs), either metallic Ag^0^ or ion form Ag^+^, are now preferred to improve the effects of Ag [25,30] and, nowadays, this nanotechnology has several biomedical applications [31]. In this context, Ag NPs might be deposed on the surface of implantable materials as a thin film with controlled density, thickness, and stability over time, in order to prevent biofilm formation on these devices [32,33,34,35,36,37]. 

Differently from antibiotics, the effects of Ag NPs are not limited to a single mechanism, but more than one event can occur simultaneously. When the Ag NPs come into conflict with prokaryotic cells, the affinity of Ag ions for sulfhydryl and thiol groups blocks the cellular respiratory chain, affecting also the cell transport system interfering with the cellular permeability [38]. Nonetheless, the positive electric charge of Ag NPs is crucial for NP interaction with the negatively charged bacterial wall and for the consequent ion penetration and ROS generation, leading to DNA and RNA damages, affecting protein synthesis and other vital processes [13,27]. Many in vitro and in vivo studies evaluated the activity of Ag NPs against microorganisms and, based on those results it could be speculated that the cytotoxic effect of Ag NPs is size-, concentration- and exposure time-dependent [39]. Indeed, as the Ag NP size decreases, not only does their stability increases, but also the surface/volume ratio, conferring them a higher ability to penetrate the cell membrane and consequently to have a higher antimicrobial potential [21,22]. Moreover, it has been demonstrated that Ag NPs interact with bacteria and fungi in a shape-dependent manner [28,40]. In recent research, Raza and colleagues verified whether the antimicrobial activity might be affected by Ag NP size and shape, concluding that the smallest spherical Ag NPs (15 to 50 nm) had a higher effect on *Pseudomonas aeruginosa* and *Escherichia coli* viability than triangular and larger spherical Ag NPs (150 nm and 30–80 nm, respectively) [40]. The different morphology might have a crucial role in the antimicrobial properties of Ag NPs, since depending on the shape, NPs might have different exposed surface areas in terms of active facets [41].

Nevertheless, the bactericidal effect of Ag depends on the NP concentration, which varies based on different bacterial susceptibility depending on the different microorganisms class. For instance, *S. aureus* requires a concentration of 33 nM to be inhibited, while *E. coli* is inhibited at lower concentrations (3.3 nM) [42]. All the listed variables cooperate to confer Ag NPs the antimicrobial effect that can be amplified by the combination of antibiotics (i.e., ciprofloxacin, imipenem, gentamycin, vancomycin, and trimethoprim), creating a synergic effect [43] 

The antimicrobial properties of Ag are recognized and well documented, and it has been described how Gram-negative bacteria are able to acquire resistances, inducing phenotypic changes to efflux transporter leading to the reduction of intracellular levels of Ag [44]. Indeed, this mechanism might involve the reduction of Ag+ to a less toxic neutral oxidation or it might be determined by the active efflux of Ag+ from the cell by either P-type adenosine triphosphatases or chemiosmotic Ag+/H+ antiporters [45]. Furthermore, it has been recently demonstrated how the exposure of Gram-negative bacteria (i.e., *E. coli* and *P. aeruginosa*) to subinhibitory concentrations of Ag NPs is able to induce production of flagellin that aggregates Ag NPs, reducing their antibacterial effect [46]. 

Finally, many concerns have been expressed by the scientific community whether Ag NPs might be a threat for eukaryotic cells surrounding the implanted devices and for tissues and organs [47]. Indeed, Ag NPs are known to be toxic to eukaryotic cells present in the bone, such as osteoblasts and osteoclasts depending on their concentration. Hence, different studies have been conducted to assess the cyto- and genotoxic potential effects of Ag NPs on human osteoblast-like cells or human mesenchymal stem cells in vitro and in vivo [48,49,50]. These analyses indicated a toxic effect strictly related to the concentration of Ag NPs that sometimes is able to trigger the generation of ROS and the subsequent oxidative damage to cellular components. However, human osteoblast-like cells demonstrated the ability to adapt in order to survive the presence of a moderate quantity (5 μg/mL) of Ag NPs [49]. 

Hence, the beneficial advantage in the use of Ag NPs lies in a wide therapeutic window; it has been demonstrated that very small concentrations of Ag NPs (35 ppb) are sufficient to induce bactericidal effects, while concentrations of 300–1200 ppb are cytotoxic to mammalian cells [27,51]. As a matter of fact, several severe side effects (e.g., argyria, leukopenia, damages of kidney, liver and neural tissue, etc.) were documented in the presence of 300 ppb of Ag NPs in the bloodstream. Conversely, blood concentrations of 56 ppb of Ag NPs can be considered biocompatible [29]. 

### 3.2. Gold

The lower toxicity compared to other inorganic NPs, ease synthesis and functionalization make gold NPs (Au NPs) optimal candidates to forestall the growth and adhesion of microorganisms [15]. Different from Ag, Au is an inert material, which lacks antibacterial properties, unless manufactured into nanostructures with rough surfaces [14]. Moreover, the antimicrobial activities of Au NPs are quite different from any other NPs because they are not induced by the generation of ROS, hypothesizing a safer use of these NPs in clinical settings [52]. 

Therefore, the MIC is significantly higher in Au NPs compared to Ag NPs to achieve antimicrobial activity. However, it has been demonstrated that a concentration of at least 120 µg/mL can have an impact on microorganisms, damaging the cell wall and interfering with cell function [53]. In particular, Au NPs have a confirmed antifungal activity depending on the effect of size and shape of particles, which modulate the amount of exposed active surface [53]. Indeed, the shape of Au NPs can be controlled during the synthesis process, and a wide variety of morphologies can be molded, such as rods, plates, branched structures, etc. [54]. Depending on the size and mostly on the shape of NPs, the surface area ratio changes accordingly. This effect was described in a study on various species of *Candida*, in which Au NPs shaped as discs (25 nm) displayed higher fungicidal activity compared to the Au NPs with a polyhedral structure (30 nm), with an MIC of 16–32 µg/mL and 32–128 µg/mL, respectively [55].

Nonetheless, Au can be functionalized to amplify the antimicrobial effect against most Gram-positive and negative bacteria. Indeed, Au NPs and nano-rods can acquire a higher bactericidal effect when conjugated and photothermally activated, for example, against *P. aeruginosa*, as demonstrated by Norman et al. [56]. In another recent study proposed by Li and colleagues [14], cationic and hydrophobic functionalized Au NPs played an active role in the inhibition of 11 of clinical MDR isolates, proposing a valid solution to impede the antibiotic resistance. The antimicrobial action explained in the study underlined that cationic and hydrophobic Au NPs promoted the interaction with the cell membrane of Gram-positive and negative bacteria, resulting in the lysis of their membranes. To enhance their antimicrobial activity, Au NPs can be also conjugated with various molecules or employed as local carriers, strengthening the antimicrobial effects due to a synergistic mechanism of action [57,58]. This specific feature not only can be exploited to carry antimicrobial molecules, but also genes or drugs taking advantage of Au NPs internalization within eukaryotic cells throughout nonspecific endocytosis. However, it has been demonstrated that once Au NPs are internalized by osteoblasts, they cannot be degraded, whereas they can be enclosed in lysosome vesicles in the cytoplasm. Although the cell viability, proliferation, and differentiation are not compromised, the Au NP internalization might interfere with proteins and consequently with the cell metabolism [59,60]. Once again, the size of Au NPs plays an important role in enhancing some cellular functions. In particular, Au NP rods (70 nm in size) markedly promoted the osteogenic differentiation of mesenchymal stem cells, while 40 nm rod-shaped Au NPs suppressed the osteogenic differentiation process [61].

Despite that a higher concentration of Au NPs is required to achieve the antimicrobial potential compared to Ag NPs, the use of Au NPs in orthopedics should not be excluded. Indeed, the conjugation of these NPs to implantable materials might be an encouraging strategy to counteract infections while promoting a safer use, as reported by Yang and colleagues, who described the Au NP-modified surface of titanium dioxide nanotubes as promising candidates for orthopedics [62]. 

### 3.3. Copper

Copper (Cu) is a necessary trace element in the human body, and, like many other transition metals, it also possesses some antimicrobial properties [63]. The metal oxide form of Cu (CuO) has been proposed because of its bactericidal effect against different microorganisms. It has been demonstrated that CuO also plays a fundamental role in the alteration of the expression and catalytic activities of some enzymes due to the reaction with protein sulfhydryls, causing damage to key proteins [64,65]. Moreover, similar to Ag NPs and Au NPs, CuO NPs are able to interact with the bacterial cell wall, causing severe damage. Indeed, the oxidative stress enhanced by the CuO NP production of ROS contributes to change the bacterial membrane permeability followed by microorganism death [19]. Furthermore, Cu ions (Cu^2+^) work as a donor/acceptor of electrons by switching between the redox states of Cu^+^ and Cu^2+^, which causes bacterial damages [66]. Similar to other transition metals, the shape, size, and microstructures are the principal factors influencing the antibacterial properties of Cu NPs [67]. It has been described that Cu NPs have a greater inhibition effect against *E. coli* compared to the Gram-positive *S. aureus* [67]. This antibacterial effect is mainly due to the interaction of Cu NPs and the bacterial cell wall which is extremely negatively charged in Gram-negative bacteria. Furthermore, the physical characteristics of CuO NPs significantly affect their antibacterial potential. Indeed, it has been demonstrated that thin CuO nanorods (Φ 5–15 nm; length 50–100 nm) have higher antibacterial effects compared to thicker CuO rods (Φ 10–40 nm; length 50–400 nm) because of their greater ability to penetrate the cell wall membrane [68].

Different from Ag NPs, CuO NPs have reduced antibacterial power, requiring higher concentrations (100–5000 μg/mL) to achieve the inhibition of microorganisms, thus raising concerns in the biosafety of CuO [69]. This intrinsic limitation can be easily bypassed by using CuO in association with other materials. For instance, CuO in combination with zinc oxide NPs demonstrated a significant inhibitory effect on oral biofilm models [70]. Moreover, the combination of NPs and titanium dioxide coatings resulted in a synergistic effect supporting the apatite formation process, biocompatibility, osteoconductivity, and antimicrobial activity [71]. This synergistic effect was also reported by Shi et al. [72] in association with hydroxyapatite that promoted bone regeneration, modulating the balance between osteoblasts and osteoclasts, together with an antimicrobial activity against *S. aureus* and *E. coli* with excellent biocompatibility. 

As a bulk material, Cu is not used for orthopedic applications. However, CuO NPs associated with materials commonly employed in prosthetic surgery or bone grafting might enhance the implant integration while preventing infections. 

### 3.4. Titanium

Titanium dioxide (TiO_2_) is frequently employed as biomaterial coating due to its antibacterial properties, non-toxicity, stability and relatively low manufacturing costs [73]. The interest in this metal arises from the intensive use of pure titanium (Ti) and titanium alloys, as most of the implantable orthopedic devices are made of these materials for their mechanical strength and resistance to fatigue, as well as bone affinity and osteoconductivity. A TiO_2_ thin film can be used to cover Ti or other alloys to preserve the features of the bulk materials and to protect their surface from threats driven by biological fluids, such as bacteria, through its crystal structure [74,75]. It has been previously reported that the exposure of material to ultraviolet (UV) light activates metal oxide, and that UV-activated TiO_2_ can damage a wide range of microorganisms such as Gram-positive and negative bacteria, fungi, algae, protozoa and viruses [76,77]. However, the activation process can be considered as a limitation in the use of TiO_2_; indeed, to enable the catalytic process, photons have to reach the material surface. Furthermore, the TiO_2_ exposure to long-wave UV (UVA) light activates metal oxide, leading to the formation of ROS, such as superoxide anion radicals and free hydroxyl, hydrogen peroxide, and singlet oxygen in aqueous solutions [78]. The limitation of TiO_2_ photo-activation together with the cytotoxicity towards multiple bone-related cells discourages the use of this NP in the orthopedic field. Also, the association of TiO_2_ NPs with orthopedic devices may lead to osteolysis, implant aseptic loosening and non-specific pain related to the alteration of bone homeostasis causing bone resorption [79]. 

### 3.5. Zinc

Zinc oxide (ZnO) NPs have multiple biological applications, as they are already used in the cosmetic and the sunscreen market for their physical properties, i.e., transparency and ability to reflect, scatter, and absorb UV radiation [80]. Furthermore, similar to other NPs, ZnO has a broad spectrum of antibacterial activity due to its physical properties. Indeed, the surface-to-volume ratio confers ZnO NPs the ability to interact with negatively charged bacteria, inhibiting their growth and adhesion [81]. The toxicity of ZnO NPs is also attributed to the release of Zn ions, which significantly influence the active transport of prokaryotic cells and the synthesis of proteins and ZnO accumulation in the cell cytoplasm [82]. Furthermore, the release of Zn ions also promotes the formation of hydrogen peroxide and ROS, another effective weapon against bacteria. However, the presence of low concentrations Zn^2+^ in the surrounding areas might induce the tolerance of bacteria to this material [81]. The bacterial resistance mechanisms to metal ions can occur at the extracellular or intracellular level by sequestering metal particles, reducing the permeability, or directly expelling the metal ions [83]. 

Among pathogens affected by ZnO activity, *S. aureus* and *S. epidermidis*, *S. pyogenes*, *B. subtilis*, and *E. faecalis* have been documented [84]. In addition, *Campylobacter jejuni* and other foodborne pathogens are affected by these NPs, demonstrating a downregulation of the virulence genes, such as cell motility, toxin production, and adhesion to host cells after treatment [85]. The concentration, shape, and size of ZnO have a determinant role in the antimicrobial activity of NPs. A recent study described how the modulation of the shape and size of ZnO enhanced the activity against bacteria [68]. In particular, the authors showed that ZnO cone NPs (Φ 80–100 nm; length 100–160 nm; 8.7 m^2^/g) had a higher activity compared to both ZnO with hexagonal shape (60–100 nm; 8.6 m^2^/g) and ZnO nanorods (Φ 30–40 nm; length 140–320 nm; 2.7 m^2^/g) because of their greater porosity and surface area [68]. 

More importantly, recent studies demonstrated how ZnO NPs have a selective toxicity to bacteria with minimal effects on human cells, the latter being more resistant to NP effects [86]. Indeed, Memarzadeh and colleagues recently described how ZnO NPs, as a coating material, inhibited *S. aureus* adhesion while promoting osteoblast growth and the consecutive osseointegration of the implant [87]. Even though a high cytocompatibility was described in the literature, it has been reported that the shape of ZnO NPs not only influences the antimicrobial efficacy but also has adverse effects against bone cells. Indeed, it has been demonstrated how spherical ZnO NPs are readily internalized by osteoblasts without impeding any cell function and how ZnO rod-shaped NPs impaired cell viability due to their physical properties [88].

Even if there is still a need to investigate all the possible side effects on other host cells (e.g., erythrocytes), the use of ZnO NPs might prevent the failure of implanted devices due to infections, but also due to aseptic loosening improving their osseointegration through enhanced bone density and mechanical properties [89].

### 3.6. Zirconium

Zirconium (Zr) is a sturdy transition metal that received special interest in different biomedical fields due to its physical features that resemble those of titanium [90]; nevertheless, bulk Zr is not currently used to produce orthopedic implants. However, Zr alloys and zirconium dioxide (ZrO_2_), commonly known as zirconia, are widely employed in orthopedics for joint replacement [91], but also in dentistry for dental crown reconstruction and dental implants [92,93] due to its good biocompatibility and high fracture strength [94]. Different from other oxides, there is currently a lot of debate about whether ZrO_2_ NPs have a toxic activity against bacteria and fungi. Jangra and colleagues [95] investigated the correlation between the morphology and physical properties of ZrO_2_ NPs and Zr complexes and antimicrobial activity against *E. coli*, *S. aureus*, *Botrytis cinerea*, *Aspergillus niger*, and three other *Aspergillus* species. In this study, it was demonstrated how the structure of ZrO_2_ NPs and Zr complexes (e.g., surface areas and specific crystal plane) might influence the activity against bacteria. Indeed, it was observed that the ZrO_2_ nanostructures had an antibacterial activity against *E. coli*, but not against *S. aureus* and fungi. Conversely, Zr complexes had an action against both *S. aureus* and *E. coli* [95]. These results were ascribed to the atomic arrangements of different exposed surfaces, and the authors concluded that ZrO_2_ NPs with the same surface areas but with different shapes showed different antimicrobial activity [95]. 

Antimicrobial activity observed against Gram-negative bacteria was also reported by others describing the inhibitory action of ZrO_2_ NPs against *P. aeruginosa* [96]. This phenomenon is probably due to the outer membrane of Gram-negative bacteria that is mainly composed of phospholipids and lipopolysaccharides, which are known to be strongly negatively charged. The bacteria’s negatively charged cell surfaces attracted NPs, enhancing the toxic their activity [96]. The modest effects of ZrO_2_ NPs have been described by Banerjee and colleagues, who underlined again the importance of NP structure and concentration that confer antimicrobial activity [97]. 

Even though studies on the antimicrobial properties of ZrO_2_ often report inconsistent results, the zirconia-nanosized modification of metal implant surfaces might support a better integration with the biological system for long-term applications. Indeed, the bioceramic nature of this material enhances the expression of integrins and the hydrophilicity of implant surfaces, promoting the adhesion of osteoblasts and subsequent bone maturation. Furthermore, a recent in vivo study demonstrated the absence of delamination and wear debris of metal implants with a nanostructured zirconia surface, along with the lack of any inflammatory and foreign body reaction [98]. These advantages make zirconia a possible candidate as an antimicrobial material for orthopedic implants with low adverse effects. 

### 3.7. Iron

Iron (Fe) is fundamental for human and animal health. Indeed, this element has an important role in oxygen transport and cellular respiration. The physiological Fe level is around 2.5–4 g in females and males, respectively, distributed in hemoglobin throughout the body [99]. Furthermore, the uptake of iron through the diet has been demonstrated to have a beneficial activity on bone mineral density [100]. 

Iron oxide NPs are currently employed in an expanding number of medical applications, from cell labeling, separation and tracking to cancer therapy [101]. Nonetheless, superparamagnetic iron oxide (SPIO) nanoparticles were proposed in clinics as long-term tracking/labeling system detectable with imaging techniques already applied for diagnostic purposes [102] or proposed to evaluate the outcome of tissue engineering strategies with magnetic resonance [103].

Iron in its bulk form is an inert material lacking antimicrobial properties [5]. Though, similar to Au, when nano-synthesized, Fe acquires antimicrobial features. 

A recent study investigated the properties of FeNPs against some Gram-negative (*Erwinia amylovora*, *Xanthomonas oryzae*) and positive bacteria (*Bacillus cereus* and Streptomyces spp.) [104]. *B. cereus* and Streptomyces spp. demonstrated a higher tolerance, displaying only growth inhibition without any bactericidal effect probably due to the thickness of the peptidoglycan membrane. It has been hypothesized that the bactericidal effect is once again mediated by the formation of ROS. In particular, superoxide, hydroxyl radicals and hydrogen peroxide might lead to cytotoxic effects not only in prokaryotic cells, but also the DNA and protein synthesis of eukaryotic cells [105]. Another recent study demonstrated the effects of Fe_3_O_4_ against Gram-negative bacteria (*E. coli*, *Serratia marcescens*, and *P. aeruginosa*) and positive bacteria (*S. aureus*) [106]. The smaller the NPs size, the larger the surface area interacting with bacteria and causing bacterial permeability leading to cellular disruption. Indeed, Fe_3_O_4_ NPs are of particular interest as antibacterial agents because during the synthesis process they can be molded with unusual crystalline morphologies with a high number of edges and corners characterized by extremely high surface areas increasing their reactivity against bacteria [107].

Furthermore, once again, it has been reported that the reduced iron species (Fe^3+^ and Fe^2+^) cause the formation of ROS altering the intracellular balance by depolymerizing polysaccharides, breaking DNA and inactivating enzymes, resulting in cell death [106]. Due to the increased use of FeNPs, many efforts are currently made to assess the nanotoxicology and the potential damages on host cells derived by the use of this metal [108,109]. Indeed, the conjugation of Fe_3_O_4_ to other materials reduces the exposed NPs, and consequently decreases the formation of ROS from the Fe_3_O_4_ surface in contact with body fluids and cells [110]. This successful strategy allowed the mineralization of the Fe_3_O_4_ NP-coated materials, and, therefore, bone orthopedic and therapeutic applications. 

## 4. Discussion

The wide range of potential applications of NPs in medicine is strictly related to their physiochemical features and surface charge along with shape, size, and concentration. However, the well-demonstrated, broad-spectrum antibacterial properties of NPs against Gram-positive and negative microorganisms make them valid candidates to fight infections in the orthopedic field, and particularly in implant-related bone infections. The major mechanisms of action of NPs are explicated by metal ion release, oxidative stress induction, or non-oxidative mechanisms. Thus, the antimicrobial activities determine the penetration and disruption of the bacterial cell, the generation of ROS and intracellular effects, i.e., interactions with DNA and proteins. 

Unfortunately, all these mechanisms also act towards cells present in the human body. This implies the need to have a deeper insight into the toxic effects of NPs, mainly related to the non-specific binding to host cells, biological fluids and the consequent accumulation in tissues and organs of living organisms. Despite several in vitro studies that demonstrate the balance between antimicrobial activities and eukaryotic cell safety, the wide range of NPs concentrations, exposure time and tested cell lineages represent the major drawback in obtaining consistent and reproducible results. More importantly, the precise investigation of NP biodistribution and pharmacokinetics is mandatory for their translatability to clinics. However, similar limitations of in vitro evaluations can be derived from studies that extensively investigated the systemic toxicity of NPs on the metabolism or immune system of rodents treated through the intravenous, dermal, subcutaneous, inhalation, intraperitoneal, and oral routes [111]. Thus, further studies should be performed to better correlate the in vitro with the in vivo effects of NPs, also concerning the synthesis process.

Indeed, the fabrication process of NPs deserves to be taken into account because it has an influence on the potential toxicity of these molecules as well as on the costs related to NP production. NPs are usually generated by chemical, physical, or biological synthesis, of which the chemical synthesis often showed cytotoxic effects due to the presence of agents used in the generation process. In contrast, physical methods (i.e., laser-synthesized NPs) could have greater success in medical applications because of the absence of residual toxic compounds [112]. Similarly, the green biosynthesis of NPs from natural products could be an alternative method to develop environmentally friendly and non-toxic NPs exploiting renewable materials [13]. The green synthesis of NPs results in a product with enhanced stability and biocompatibility, due to the possibility to vary the range of sizes, shapes, and compositions of biosynthesized NPs with minor use of hazardous chemicals [113]. Several biological materials such as algae, as well as leaves, roots, and tubers, have demonstrated antibacterial power [114]. For example, green tea extract from *Camellia sinensis* leaves was exploited to produce Ag NPs [115], while *Mentha piperita* leaf extract included both Au and Ag NPs with antibacterial properties against both Gram-positive and negative bacteria [116]. The green synthesis of antibacterial NPs is also a cost-effective strategy that allows the production of several transition metal NPs without resorting to the traditional chemical synthesis, contributing to the protection of the ecosystem [117]. The use of maize waste materials, for example, is a profitable and eco-friendly strategy for NP synthesis.

It is implicit that currently the in vitro and in vivo studies furnish the results of NPs in their free form and that their conjugation with implant or construct surfaces can modify either the cultured cells or the host response. Indeed, one of the main strategies to be employed to diminish the potential toxicity of NPs is their use as coating materials for orthopedic implants. In fact, linking NPs to the surface of implant devices as well as to various natural or polymer structures could generate suitable biocompatible materials able to enhance NP stability, biological fluid dispensability, and biocompatibility. To spatially confine and stabilize NPs to avoid their aggregation due to their surface charge and electric potential, capping agents (e.g., citrate, chitosan, polyethylene glycol (PEG), and hydroxyapatite, etc.) have been proposed. Ag NPs stabilized with citrate or chitosan reported an enhanced capability in killing bacteria compared to Ag NPs alone, mainly due to an increased production of Ag ions from the NPs [118]. Conversely, Ag NPs linked to hydroxyapatite were able to release a large number of ions able to counteract bacterial colonization at the very beginning of their release [119]. Similarly, PEGylated NPs led to a lower antimicrobial activity, thus reducing their potential use for this purpose [118]. Whilst this approach could represent an advantage in the biosafety of NPs, their antimicrobial properties and uncontrolled ion release could be affected when NPs are stably linked to the surface of orthopedic implant materials. 

Regardless of their form, the antibacterial efficacy of NPs either free or conjugated with implantable devices relies on the balance between risks and benefits, as for any other drugs. In this context, the assessment of a therapeutic window is mandatory for a more conscientious use of nanoparticles in clinics. Indeed, the therapeutic dose should be sufficiently high to guarantee cytotoxic effects against prokaryotic cells, but not to eukaryotic cells. Indeed, side effects related to the use of NPs are not only locally confined to surrounding cells, but major systems (i.e., respiratory, neurological and circulatory) might also be affected [120]. 

Furthermore, the concentration should be sufficiently low to guarantee the safety while discouraging the development of the antimicrobial resistance. Indeed, the plasticity of the bacterial genome confers to microorganisms the ability to tolerate the stressful stimuli caused by the presence of a few ions or NPs. This event might result in the modification of phenotypic and genotypic features of bacteria, such as the ability to repair DNA damages or to produce enzymes able to counteract the nitrosative stress [121]. 

While it could be very easy to measure out the levels of free NPs in a living organism, the detection of ions or molecules released from the surface of implants might be demanding and subjected to the host physio- or pathological microenvironment [122]. Furthermore, the release of the aforementioned particles from implant devices cannot be controlled over time, thus resulting in chronic NP exposure and intake.

Nonetheless, the nano-sized modification of implant surfaces must be analyzed for corrosion resistance in order to guarantee some tribological characteristics. Metal implants are protected from corrosion by a surface oxide layer; however, mechanical stress may defeat this protection, thus leading to the release of detrimental metallic particles. Indeed, it is well-known that any metal implant undergoes mechanisms able to produce nanoscopic metal wear debris and corrosion products (25–36 nm), causing the elevation of ion levels correlated to local and systemic adverse effects (i.e., inflammation, hypersensitivity, osteolysis, etc.) [123]. Even if a milder antimicrobial and cytotoxic activity of TiO_2_ compared to Ag has been demonstrated, the need to expose the material to UV light in order to activate the metal oxide limits the possible use of TiO_2_ NPs conjugated to orthopedic devices. Although titanium alloys are considered inert, they can be a source of TiO_2_ NPs from bone implants that have been recently demonstrated to impair bone formation and to interfere with bone resorption at the site of repair, thus leading to aseptic loosening of implants and pain [124]. Differently, Zr demonstrated promising features for its use in the orthopedic field through the ability to support a better integration with biological systems without the generation of wear debris from the surface of nanostructured zirconia implants. 

Among several antibacterial NPs, the noble metals, like Ag and Au, have gained much attention. Indeed, nanomaterials are increasingly an integral part of orthopedic implants and scaffolds. The main advantage of such solutions is the ability to manipulate the properties of biomaterial surfaces on a nanometric scale. Thanks to Ag antimicrobial properties *per se* or as NPs, market products containing Ag NPs are used for wound care (Acticoat) or catheters (I-Flow SilverSoaker Nanosilver), as reported in “The global nanomaterials market, 2010–2025”. In the patent by Yan and colleagues (US 6379712 B1), the preparation of granules coated with Ag NPs acting as an antibacterial and antifungal agent has been described, thus opening the use of these NPs as a component of various types of preparations. The use of Ag NPs to impede the attachment of bacteria to the surface of dental implants is also known (Patent US 2007/0293799 A1) [79]. However, nowadays no clinical trials evaluating the use of transition metal NPs in orthopedics exist, only a few trials are currently investigating the use of Ag, TiO_2_, and Zn NPs in dentistry [125]. From this analysis it appears that the need for the commercial use of NPs is clearly faster than the response of the research on the toxicity of these nanomaterials [126]. Indeed, NP aggregation due to their high surface energy and toxic nature limits their use. Thus, attention should be given to the interaction of NPs and biological fluids, which supports their aggregation and reduces the possibility to correctly evaluate the NP concentration within organisms. Concrete examples of the underestimated toxicity of NPs led to treatment of burned patients with ionic Ag, causing reactions of hypersensitivity [127]. In orthopedics, some studies reported concentrations of silver 1000 times the serum baseline that led to neuropathy and muscle paralysis in patients who underwent total hip arthroplasty and were treated with silver-impregnated cement [128,129]. To the best of our knowledge, only one study evaluated in vivo the effects of Ag NPs as a titanium-based implant coating to prevent staphylococcal biofilm in orthopedics [130]. In the study, the medullary cavity of rat femur was inoculated with *S. aureus* ATCC 35984 and Ag NPs immobilized or pure titanium K-wire was inserted into the cavity. Ag NPs were found to reduce the risk of implant-associated peri-prosthetic infection. 

On the other hand, Au has a good balance between its antimicrobial activity in the form of NPs and cytocompatibility, because of the inability to generate ROS. Furthermore, recently, a new class of drugs for rheumatoid arthritis has been developed, exploiting the Au NP ability to invade macrophages and stop them from producing inflammation without killing them. The researchers found that by reducing gold into NPs (50 nm), more gold was absorbed into the cells, with much less toxicity in the case of rheumatoid arthritis [131]. Again, Au NPs have been widely used in imaging and diagnosis of many diseases [132], or as intravenous contrast agents for imaging and noninvasive detection of lung cancer and many other topics [133]. Even if the use of Au NPs is not yet approved by the US Food and Drug Administration, there are clinical trials currently evaluating the use Au NPs in many different clinical fields, as aforementioned in immunotherapy (e.g., rheumatoid arthritis) and imaging [125,134]. This scenario reports a debatable situation. The controversy is well explained in the findings of Villiers et al., who analyzed the viability of murine dendritic cells incubated in the presence of Au NPs, showing that these particles are not cytotoxic, even at high concentrations. However, the analysis of the cells revealed significant amounts of Au NPs in endocytic compartments and a higher secretion of cytokines, thus demonstrating a potential adverse event in the immune response [135]. Although in vitro tests are useful to screen compounds and to recognize effects induced on cells, they may not be sufficient to define safe exposure limits.

Finally, it must be taken into account that excessive levels of essential trace elements are harmful to living organisms, despite their vital role [13], thus discouraging the use of Cu, Zn, and Fe for medical purposes. Indeed, these elements are known to have a significant influence on physiological mechanisms involved in cellular metabolism, immune function, wound healing, protein synthesis and acting as antioxidant [136]. An imbalance of the physiological presence of these trace elements could lead to the development of severe human diseases, including cancer, hepatic and neurodegenerative diseases. In particular, Fe excess has been demonstrated to be associated with the pathogenesis of Alzheimer and Parkinson’s diseases interacting with amyloid β-peptides. An exaggerated abundance of Cu impairs the mitochondrial respiratory chain, affecting the calcium retention capacity of cardiomyocytes [137]. Finally, Zn is the second most abundant transition metal in organisms after Fe, and in contrast to Fe and Cu, it is redox-inert despite numerous findings that demonstrated a pro-oxidant role causing cell death [138,139]. An excessive absorption of Zn suppresses physiological Fe and Cu intake, despite that Zn accumulation is very rare [136]. 

According to this premise, nowadays, the research community perfectly responded to the antibacterial advantages of using NPs, in particular, Ag NPs, but the unknown-or better-incomplete known about the toxic effects related to these nanomaterials makes it necessary to conduct further research on the toxicity of NPs in living organisms. The basic question to be addressed is: how toxic are NPs at the potential concentrations at which they might be used for therapeutics? In addition, at present, even if several reports have been published, the problem remains basically unsolved. Thus, evaluation of the safety of nanomaterials is mandatory to balance the risks and benefits for their successful development and translatability. Specifically, in the case of use of NPs as antimicrobial coatings on implant devices, the question of their toxicity becomes challenging due to the great difficulty to evaluate the released amount after implantation in living organisms. Moreover, an undervalued aspect is the clearance of NPs from the body after their therapeutic effect is concluded. In this respect, it is also important to state that there is a diversity between cytotoxicity and cellular damage. Indeed, NPs without or with poor cytotoxicity may determine cellular damage. On the basis of this scenario, a common and standardized approach to investigate the effective level of toxicity in different experimental setups is urgently required, starting from the physical and chemical properties of NPs, through the in vitro results to their effects in vivo.

Despite the promising future of Ag- and Au NPs as antimicrobial strategies in orthopedics, there are many essential issues to be addressed, such as NP stabilization onto the implant devices, the therapeutic window within which NPs can be employed in the absence of side-effects, the long-term fate and effects of NPs in the organisms to be used in humans without risks. Considering this aspect, a complete preclinical evaluation of NP safety, toxicology and kinetics should be dutifully verified in in vivo models.

Nevertheless, although the toxicity and persistence of NPs within filter organs are not well verified, nowadays, several basic research on NP development and potential use is published in top journals by overemphasizing NP potentialities, but without any existing potential of translation in clinics. In fact, no NP-based antibacterial drugs have currently achieved FDA approval [100], despite the emergence of a variety of nano-functionalized materials. 

Thus, the imbalance between advantages and disadvantages in using NPs raises this question: why is there this trend? Furthermore, the more complex the nanomaterials, and the efforts to make them more biocompatible, the higher will be the costs of their production with much reduced potential for their commercialization. This is mainly true in the case of orthopedic infections compared to oncologic or lethal diseases, in which NPs could represent an innovative weapon in the absence of other strategies.

In this context, the main purpose of this review article is to analyze the wide panorama of transition metal-derived NPs of particular interest as possible coating materials for prosthetic implants in order to fight or prevent infections. Rather than making a systematic review of the most recent work, here we highlighted how the non-standardized use of metal NPs in research studies prevents the possibility of reaching a general conclusion regarding the effective safety of these NPs. Moreover, this analysis wants to caution the readers regarding regulations advancing the development and use of NPs. Hopefully, future work in vivo and clinical trials will support and regulate the use of nano-coatings to guarantee a safer use of this promising approach against antibiotic-resistant microorganisms.
